# Perforation in Typhoid Fever

**Published:** 1899-11-04

**Authors:** 


### PERFORATION IN TYPHOID FEVER.

In recording a case of typhoid fever in which perforation of the cæcum took place, and in which operation was followed by recovery, Dr. Herbert Hawkins and Mr. Thurston[1] say that in severe cases of typhoid fever it is often impossible to recognise the occurrence of perforation quickly and certainly, but when it can be recognised it is clear that surgical treatment affords an appreciable chance of recovery. Of 105 cases recorded by the several observers mentioned, 22 resulted in recovery. Success appears to depend mainly upon the time at which surgeons can operate, for figures seem to show that the cases which are dealt with in the second twelve hours after perforation are the most favourable, this time corresponding to the recovery from the initial shock.

[1] Lancet, Oct. 14.

